# X-linked SBMA model mice display relevant non-neurological phenotypes and their expression of mutant androgen receptor protein in motor neurons is not required for neuromuscular disease

**DOI:** 10.1186/s40478-023-01582-1

**Published:** 2023-06-02

**Authors:** Anastasia Gromova, Byeonggu Cha, Erica M. Robinson, Laura M. Strickland, Nhat Nguyen, Mai K. ElMallah, Constanza J. Cortes, Albert R. La Spada

**Affiliations:** 1 Departments of Pathology and Laboratory Medicine, Neurology, and Biological Chemistry, University of California Irvine, Irvine, CA 92697 USA; 2 Department of Neurology, Duke University, Durham, NC 27710 USA; 3 Division of Pulmonary Medicine, Department of Pediatrics, Duke University, Durham, NC 27710 USA; 4 School of Gerontology, University of Southern California, Los Angeles, CA 90089 USA; 5 Department of Biological Chemistry, University of California Irvine, Irvine, CA 92697 USA; 6 UCI Institute for Neurotherapeutics, University of California Irvine, Irvine, CA 92697 USA

## Abstract

X-linked spinal and bulbar muscular atrophy (SBMA; Kennedy's disease) is a rare neuromuscular disorder characterized by adult-onset proximal muscle weakness and lower motor neuron degeneration. SBMA was the first human disease found to be caused by a repeat expansion mutation, as affected patients possess an expanded tract of CAG repeats, encoding polyglutamine, in the androgen receptor (*AR*) gene. We previously developed a conditional BAC fxAR121 transgenic mouse model of SBMA and used it to define a primary role for skeletal muscle expression of polyglutamine-expanded AR in causing the motor neuron degeneration. Here we sought to extend our understanding of SBMA disease pathophysiology and cellular basis by detailed examination and directed experimentation with the BAC fxAR121 mice. First, we evaluated BAC fxAR121 mice for non-neurological disease phenotypes recently described in human SBMA patients, and documented prominent non-alcoholic fatty liver disease, cardiomegaly, and ventricular heart wall thinning in aged male BAC fxAR121 mice. Our discovery of significant hepatic and cardiac abnormalities in SBMA mice underscores the need to evaluate human SBMA patients for signs of liver and heart disease. To directly examine the contribution of motor neuron-expressed polyQ-AR protein to SBMA neurodegeneration, we crossed BAC fxAR121 mice with two different lines of transgenic mice expressing Cre recombinase in motor neurons, and after updating characterization of SBMA phenotypes in our current BAC fxAR121 colony, we found that excision of mutant AR from motor neurons did not rescue neuromuscular or systemic disease. These findings further validate a primary role for skeletal muscle as the driver of SBMA motor neuronopathy and indicate that therapies being developed to treat patients should be delivered peripherally.

**Supplementary Information:**

The online version contains supplementary material available at 10.1186/s40478-023-01582-1.

## Introduction

X-linked spinal and bulbar muscular atrophy (SBMA, Kennedy's Disease) is an inherited neuromuscular disorder characterized by lower motor neuron degeneration [1]. SBMA is caused by repeat expansions of CAG trinucleotides, encoding the amino acid glutamine (Q), in the first exon of the human androgen receptor (*AR*) gene [2], and is therefore one of nine neurodegenerative disorders that result from polyglutamine (polyQ) proteins [3]. We previously set out to determine the cellular basis of SBMA disease pathogenesis and to define key aspects of SBMA molecular pathology by focusing on polyQ-expanded AR transcriptional dysregulation. To achieve these goals, we created a novel mouse model of SBMA over one decade ago by generating bacterial artificial chromosome (BAC) transgenic mice with a floxed first exon (i.e., the BAC fxAR121 line) to permit cell-type specific excision of the AR transgene. After characterizing these mice and validating their utility for conditional termination of polyQ-AR transgene expression, we crossed BAC fxAR121 mice with Human Skeletal Actin (HSA)-Cre mice, and documented in 2014 that excision of the AR121Q transgene from skeletal muscle prevented development of systemic and neuromuscular SBMA disease phenotypes, revealing a crucial role for muscle expression of mutant polyQ-AR in SBMA motor neuron degeneration [4]. We also produced antisense oligonucleotides (ASOs) directed against AR, and upon peripheral delivery of anti-AR ASOs to BAC fxAR121 mice and AR 113Q knock-in mice, we demonstrated that peripheral suppression of polyQ-AR expression rescued motor deficits, reversed alterations in muscle gene expression, and markedly extended lifespan in both SBMA mouse models [5]. These provocative findings implicate skeletal muscle as a key site for SBMA motor neuron disease pathogenesis.

Although our prior work has demonstrated a central role for skeletal muscle in the pathogenesis of SBMA motor neuron degeneration, the contribution of motor neuron expression of mutant polyQ-AR to the disease process has remained controversial. Here we returned to our BAC fxAR121 mouse model to directly examine the contribution of polyQ-AR motor neuron expression to established SBMA disease phenotypes by crossing BAC fxAR121 mice with two lines of Cre recombinase expressing transgenic mice with different motor neuron-specific promoters: VChAT and Hb9. However, before initiating this experiment, we observed reduced disease severity in our colony of BAC fxAR121 and sought to carefully characterize defining features of the milder phenotype and to evaluate potential reasons for this change. In addition to utilizing the BAC fxAR121 mice to examine the contribution of motor neuron expression of polyQ-AR to SBMA neurodegeneration, we have also become interested in understanding a set of important non-neuronal disease phenotypes recently described in human SBMA patients: non-alcohol-associated fatty liver disease and cardiac disease [6–9]. As BAC fxAR121 mice (and all other widely used SBMA mouse models) succumb to disease by one year of age, we focused our studies of these non-CNS disease processes in BAC fxAR121 mice crossed with HSA-Cre mice, as we hypothesized that “muscle-rescued” male BAC fxAR121; HSA-Cre bigenic mice with extended lifespan would allow these systemic phenotypes to emerge. Here, for the first time, we report the existence of non-alcohol-associated fatty liver disease and cardiomyopathy in a mouse model of SBMA. Finally, characterization of the progeny of BAC fxAR121 mice crossed with either VChAT-Cre or Hb9-Cre mice revealed that male BAC fxAR121 mice lacking expression of polyQ-AR in motor neurons do not exhibit any amelioration of their disease phenotypes. In the case of the Hb9-Cre cross, where excision of the human AR transgene was extremely highly efficient, we actually observed worsened respiratory muscle function and shortened lifespan in male BAC fxAR121; Hb9-Cre bigenic mice, highlighting the potential importance of normal AR protein function in motor neurons in certain physiological settings.

## Results

### Detection and characterization of disease severity change in BAC fxAR121 mice

**Fig. 1:**
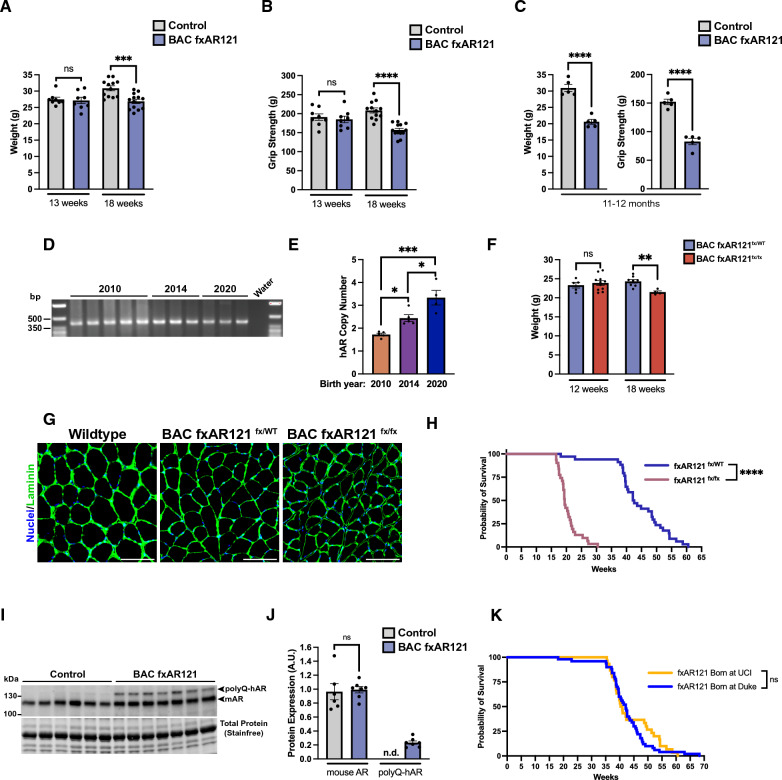
Disease progression and transgene expression in contemporary cohorts of male BAC fxAR121 mice. **A** Weight of BAC fxAR121 and wildtype male mice at 13 and 18 weeks, *n* = 8–13 mice/group. Two-tailed *t*-test, ***P < 0.001. **B** Grip strength of BAC fxAR121 and wildtype male mice at 13 and 18 weeks, *n* = 8–13 mice/group. Two-tailed t-test, ****P < 0.0001. **C** Terminal weight and grip strength metrics in 11–12 month-old BAC fxAR121 mice and controls, *n* = 5 mice/group. Two-tailed *t*-test, ****P < 0.0001. **D** Analysis of genomic polyQ length by PCR using DNA from mice born in 2010, 2014, and 2020. **E** Transgene (hAR) copy number analysis using genomic DNA from BAC fxAR121 mice born in 2010, 2014, and 2020, *n* = 4–5 mice/group. One-way ANOVA with post-hoc Tukey test, *P < 0.05, ***P < 0.001. **F** Weight of heterozygous (fx/WT) and homozygous (fx/fx) BAC fxAR121 mice at 12 and 18 weeks of age, *n* = 4–11 mice/group. Two-tailed t-test, **P<0.01. **G** Representative images of immunostaining performed on cross-sections of quadriceps muscle from 20 week-old male wildtype, BAC fxAR121 heterozygous (fx/WT), and BAC fxAR121 homozygous (fx/fx) mice. Laminin marking the outside of individual muscle fibers (green); nuclei (blue). Scale bar = 100 µm. **H** Kaplan–Meier survival analysis of heterozygous and homozygous BAC fxAR121 mice, *n* = 31–34 mice/group. Median survival of heterozygous BAC fxAR121^fx/WT^ mice was 42.4 weeks, while median survival of homozygous BAC fxAR121^fx/fx^ mice was 19.4 weeks. Log-rank Mantel-Cox test, ****P < 0.0001. **I** Western blot analysis of AR protein expression in quadriceps muscle of 15-week-old male BAC fxAR121 mice and wildtype controls born in 2019–2020. **J** Densitometry analysis of AR protein expression. Endogenous mouse AR (**I**, lower band) and polyQ human AR (**I**, upper band) are quantified separately and normalized to total protein, *n* = 6 mice/group. Two-tailed *t*-test, *P* = n.s. **K** Kaplan–Meier survival analysis of BAC fxAR121 mice born at Duke University between 2017 and 2020 compared to those born at UC Irvine between 2020 and 2022, *n* = 30–50 mice/group. Median survival of BAC fxAR121 mice born at Duke was 42.1 weeks, and median survival of mice born at UC Irvine was 40.6 weeks. Log-rank Mantel-Cox test, *P* = n.s. Error bars = s.e.m

To definitively assess the role of skeletal muscle expression of mutant polyQ-expanded AR in SBMA motor neuron degeneration, we developed a novel BAC transgenic mouse model carrying the entire human AR gene in proper genomic context with adjacent regulatory elements, and introduced a 121 CAG repeat expansion and loxP sites flanking the first exon [10]. The resulting line of ‘BAC fxAR121’ mice were then crossed with human-skeletal actin (HSA) Cre-recombinase transgenic mice to derive BAC fxAR121; HSA-Cre bigenic mice lacking expression of polyQ-AR in skeletal muscle, which was sufficient to prevent onset of neuromuscular disease and early death [4]. As reported in the initial description of the BAC fxAR121 mice, onset of neuromuscular disease emerged at ~ 8 weeks of age, and by 13 weeks of age, BAC fxAR121 male mice displayed reduced body weight and markedly decreased grip strength [4]. Disease progression was rapid, with ~ 50% of male BAC fxAR121 mice succumbing by 18 weeks of age, and 100% of male BAC fxAR121 mice dead by 32 weeks of age. Since its creation in 2009, the BAC fxAR121 line has been maintained as a congenic strain on the C57BL/6 J strain background and while the phenotype remained constant for its first 7 years of use, we noted that disease onset became delayed, and the rate of disease progression slowed in 2016 about two years after publication of the initial description. This amelioration of disease phenotype has stably continued until the present such that current cohorts of male BAC fxAR121 mice do not exhibit detectable disease phenotypes until 18 weeks of age (Fig. 1A-B). In terms of disease progression, male BAC fxAR121 mice do not display dramatic weight loss and greatly reduced grip strength until 11 – 12 months of age for most affected individuals (Fig. 1C). Despite disease becoming much milder by comparison, male BAC fxAR121 still show significantly reduced lifespan with most mice living beyond 40 weeks of age, but very few surviving past one year of age.

### Reduced disease severity stems from decreased transgene expression, not repeat contraction

Although the AR CAG repeat is among the most stable of expanded CAG repeat mutations with small contractions and small expansions equally common among intergenerational transmission in human patients and carriers [11], one obvious explanation for reduced disease severity in the BAC fxAR121 mice would be a contraction of the 121 CAG repeat expansion. To assess CAG repeat size, we performed PCR amplification of a fragment containing the repeat using primers flanking the *AR* polyQ tract, and using genomic DNA isolated from mice born in 2020 and from archival tissue from mice born in 2010 and 2014, we documented that the CAG-polyQ repeat has remained stable since these mice were originally characterized (Fig. 1D). As reduced expression of a randomly inserted transgene over time has often been observed in numerous different mouse models [12, 13], we wondered if a reduction in the number of tandemly inserted copies of the BAC transgene might account for the reduced severity observed since 2016. However, when we performed quantitative PCR to determine copy number, we found that human *AR* copy number has actually increased over time from ~ 2 in 2010 mice to ~ 3 in 2020 mice, relative to endogenous mouse *Tert* (Fig. 1E). To further clarify the situation with the current colony of BAC fxAR121 mice, we took advantage of the fact that the BAC fxAR121 transgene is inserted into an autosome and so we bred mice to homozygosity. We then compared the disease phenotypes of homozygous male BAC fxAR121 mice with heterozygous male BAC fxAR121 mice. We noted that homozygous male BAC fxAR121 mice displayed accelerated weight loss and muscle atrophy at 18 weeks of age (Fig. 1F-G), and exhibited markedly reduced lifespan with disease severity that was roughly twice as severe as heterozygous male BAC fxAR121 mice (Fig. 1H), as expected. To further evaluate the expression level of human AR in the current BAC fxAR121 colony, we performed immunoblot analysis on quadriceps muscle and compared human AR protein expression to endogenous mouse AR protein expression (Fig. 1I), as we have done previously [4]. We found that the ratio of human AR protein expression to mouse AR protein expression is ~ 0.25 (Fig. 1J), which is much lower than initially reported in 2014, when we documented that human AR transgene expression at the protein level was roughly comparable to endogenous mouse AR protein expression [4]. These results confirmed that the explanation for reduced disease severity in the current BAC fxAR121 colony is decreased expression, likely due to genomic silencing of inserted transgene expression, for which various molecular mechanisms have been described (reviewed in [14]). Importantly, as our animal colony has been relocated to different institutions, we compared survival of animals while at Duke University (2017–2020) to those at UC Irvine (2020-present) and found no difference (Fig. 1K), indicating that the phenotype has been stable after the shift in human AR transgene expression that occurred shortly after the initial report.

### Rescued male BAC fxAR121; HSA-Cre mice do not develop neuromuscular disease

**Fig. 2:**
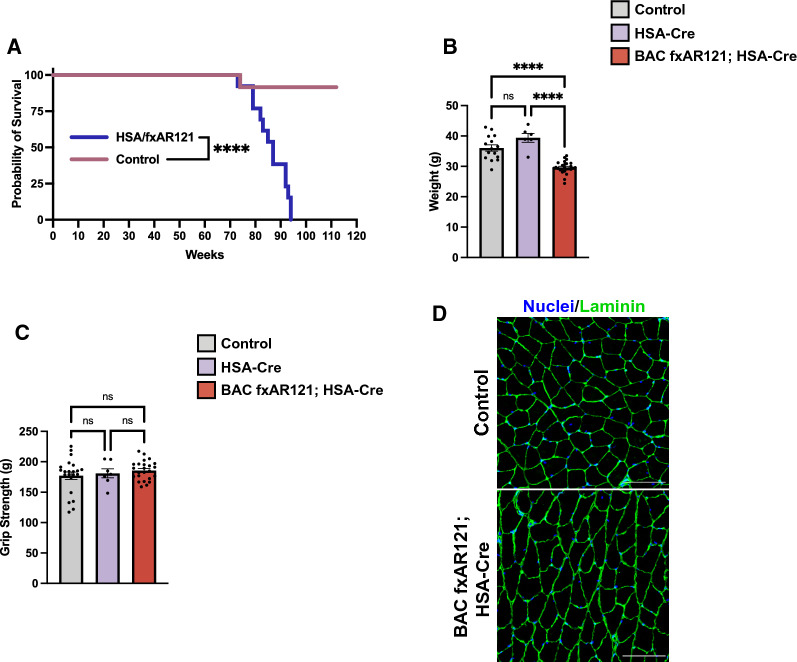
Male BAC fxAR121; HSA-Cre bigenic mice have a reduced lifespan despite lack of neuromuscular deficits. **A** Kaplan–Meier survival analysis of male BAC fxAR121; HSA-Cre and controls (wildtype or HSA-Cre only), *n* = 12–13 mice/group. Median survival of BAC fxAR121; HSA-Cre mice is 20 months/87 weeks. Only one control animal died during this time; all other control animals were euthanized after 26 months. Log-rank Mantel-Cox test, ****P < 0.0001. **B**, **C** Weight (**B**) and grip strength (**C**) metrics of aged 16–20 month-old BAC fxAR121; HSA-Cre mice compared to wildtype and HSA-Cre only controls, *n* = 6–26 mice/group. One-way ANOVA with post-hoc Tukey test, ****P < 0.0001. **D** Representative images of immunostaining performed on cross-sections of gastrocnemius muscle from 18 to 20-month-old BAC fxAR121; HSA-Cre mice and wildtype controls. Laminin (green) marks the outside of individual muscle fibers; Nuclei (blue). Scale bar = 100 µm. Error bars = s.e.m

We have previously reported that expression of human polyQ-AR protein in skeletal muscle is required for the development of disease phenotypes in the original, more rapidly progressive BAC fxAR121 mouse model of SBMA. We demonstrated the necessity of skeletal muscle expression by crossing BAC fxAR121 mice with HSA-Cre recombinase expressing mice and documenting that male BAC fxAR121; HSA-Cre bigenic mice do not display neuromuscular or systemic disease phenotypes nor suffer premature death [4]. Although neuromuscular disease is a key feature of SBMA in human patients, there has been growing recognition of serious systemic disease phenotypes occurring outside the CNS. To better understand the nature of these disease phenotypes and also to determine if long-term expression of mutant polyQ-AR in CNS motor neurons eventually affects neuromuscular function, we derived male BAC fxAR121; HSA-Cre bigenic mice, and observed a marked extension in lifespan well beyond 40 – 60 weeks in singly transgenic BAC fxAR121 mice (Fig. 2A). However, we noted that male BAC fxAR121; HSA-Cre bigenic mice do not exhibit a normal lifespan, as they die beginning at 78 weeks of age (18 months) and all succumb by ~ 95 weeks of age (22 months), unlike male non-transgenic or singly transgenic HSA-Cre littermates (Fig. 2A). When we monitored weight loss and grip strength in aged male BAC fxAR121; HSA-Cre bigenic mice in comparison to male non-transgenic or singly transgenic HSA-Cre littermates, we observed significantly reduced weight in aged male BAC fxAR121; HSA-Cre bigenic mice, but without any deficit in grip strength (Fig. 2B-C). Importantly, cross sections of gastrocnemius muscle from end-stage male BAC fxAR121; HSA-Cre bigenic mice were indistinguishable from non-transgenic littermate controls (Fig. 2D), indicating that the neuromuscular system remains intact and that the decreased weight in male BAC fxAR121; HSA-Cre bigenic mice could not be attributed to muscle atrophy. By tracking body weight over time for individual mice, we determined that male BAC fxAR121 mice steadily lose weight due to muscle atrophy until they ultimately die, and control mice (either non-transgenic or HSA-Cre only) gain weight over time due to the natural accumulation of fat during aging, but BAC fxAR121; HSA-Cre mice remain roughly the same weight throughout their lifespan (Additional File 1: Figure S1A). Moreover, upon dissection, aged male BAC fxAR121; HSA-Cre mice showed an absence of white adipose fat deposits (Additional File 1: Figure S1B). These findings suggest that altered systemic metabolic processes likely underlie the inability of BAC fxAR121; HSA-Cre mice to accumulate fat as they age.

### Rescued male BAC fxAR121; HSA-Cre mice display prominent hepatic and cardiac disease

**Fig. 3:**
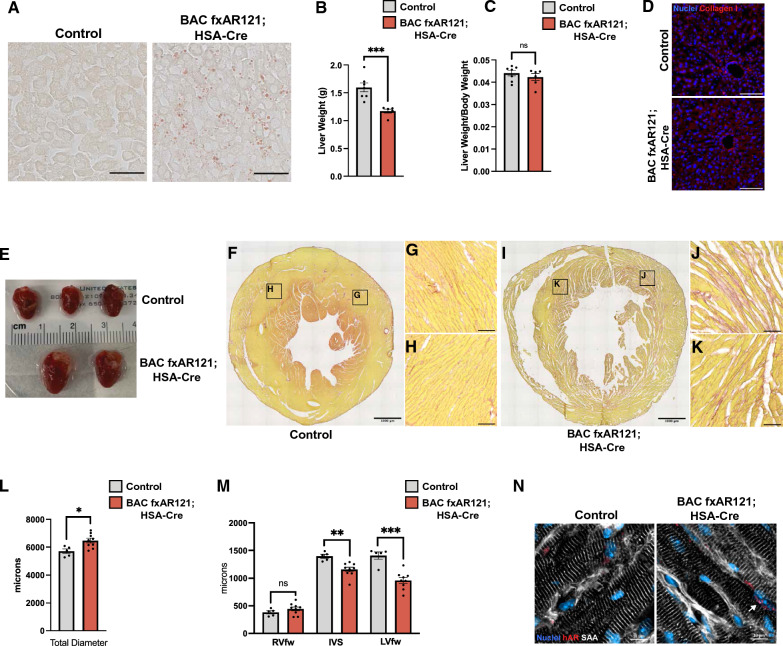
Emergent hepatic and cardiac phenotypes in aged BAC fxAR121; HSA-Cre mice. **A** Oil Red O staining of lipid droplets performed on liver sections from 18 to 20 month-old BAC fxAR121; HSA-Cre mice and controls, Scale bar = 50 µm. **B**, **C** Whole liver weights of 18–20 month-old BAC fxAR121; HSA-Cre mice and controls in absolute values (**B**) and normalized to body weight (**C**), *n* = 6–7 mice/group. Two-tailed *t*-test, ***P < 0.001. **D** Immunostaining of liver sections for Type I Collagen. Scale bar = 100 µm. **E** Dissected hearts from 18 to 20 month-old male BAC fxAR121; HSA-Cre and control animals showing the drastic increase in heart size for bigenic SBMA mice. **F**–**K** Picrosirius red staining performed on cross-sections of hearts from 18 to 20 month-old WT littermate control mice (**F**–**H**) and BAC fxAR121; HSA-Cre mice (**I**–**K**), showing thinning of the left ventricle (**F**, **I**: Scale bar = 1000 µm), with higher magnification insets showing cardiac fibrosis (**G**, **H**, **J**, **K**: Scale bar = 100 µm). **L** Measurement of total heart diameter at the mid-level. Two-tailed *t*-test, *P < 0.05. **M** Thickness measurements of the right ventricular free wall (RVfw), interventricular septum (IVS), and left ventricular free wall (LVfw). Two-tailed t-test, **P < 0.01, ***P < 0.001. **N** Representative immunostaining of human AR aggregates in hearts from 18 to 20 month-old BAC fxAR121; HSA-Cre mice and controls. Sarcomeric alpha-actinin (white), human AR (red), nuclei (blue). White arrow denotes a cardiac nucleus containing polyQ-AR aggregates. Scale bar = 10 µm. Error bars = s.e.m

Two important systemic disease phenotypes which are increasingly recognized in human SBMA patients are fatty liver disease and cardiac dysfunction. Indeed, recent studies indicate that many SBMA patients qualify for a diagnosis of non-alcohol-associated fatty liver disease [6, 7]. When we examined liver histology in aged male BAC fxAR121; HSA-Cre bigenic mice by performing Oil Red O staining, we noted a marked accumulation of lipid droplets both inside and in between hepatocytes (Fig. 3A). We also found that livers from male BAC fxAR121; HSA-Cre bigenic mice were hypotrophic, as they weighed less than livers from male non-transgenic or Cre-recombinase singly transgenic littermates (Fig. 3B). However, when normalized to body weight, there was only a modest decrease in liver mass in male BAC fxAR121; HSA-Cre bigenic mice (Fig. 3C). Next, we sought to determine the severity of liver pathology by assessing fibrosis, a result of chronic hepatocyte damage. Livers of aged BAC fxAR121; HSA-Cre mice did not show excess accumulation of Type I Collagen (Fig. 3D), suggesting that liver disease in these SBMA model mice is only mild to moderate. In addition to non-alcohol-associated fatty liver disease, SBMA patients can develop abnormal cardiac rhythms and may exhibit changes indicative of cardiomyopathy [8, 9]. To determine if cardiac abnormalities are present in male BAC fxAR121; HSA-Cre bigenic mice, we performed necropsies on end-stage mice and noted dramatic cardiomegaly in SBMA model mice compared to male non-transgenic or singly transgenic HSA-Cre littermate controls (Fig. 3E). Picrosirius red staining of short axis cross-sections of hearts from end-stage BAC fxAR121; HSA-Cre mice revealed marked fibrosis, particularly in the cardiac muscle of the left ventricle (Fig. 3F-K). Using sections that corresponded to the mid-level of the heart (based on the presence of papillary muscles), we measured the total diameter and thickness of the right ventricular free wall (RVfw), interventricular septum (IVS), and left ventricular free wall (LVfw). The total diameter of hearts from BAC fxAR121; HSA-Cre was significantly increased (Fig. 3L), and the thickness of the IVS and LVfw were markedly thinned (Fig. 3M), suggestive of dilated cardiomyopathy. Immunostaining of cardiac sections from male BAC fxAR121; HSA-Cre bigenic mice with an anti-AR antibody revealed the presence of AR aggregates in cardiomyocyte nuclei (Fig. 3N). These studies indicate that when male BAC fxAR121; HSA-Cre bigenic mice are rescued from neuromuscular disease which would have led to premature death, such mice go on to develop prominent cardiac and hepatic disease phenotypes, which resemble abnormalities detected in human SBMA patients.

### Excision of polyQ-AR expression from motor neurons does not improve SBMA disease phenotypes

**Fig. 4:**
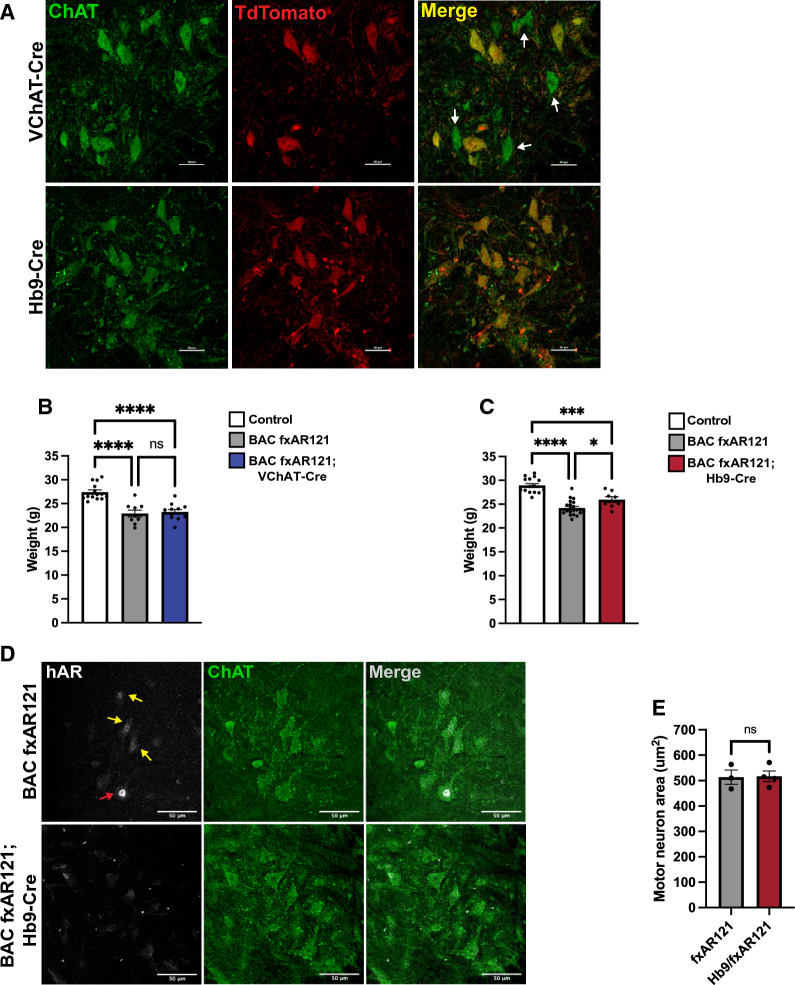
Motor neuron-rescued SBMA mice do not display improvements in disease phenotypes. **A** Representative images of spinal cord sections from VChAT-Cre; TdTomato and Hb9-Cre; TdTomato mice stained for acetylcholine transferase (ChAT), a marker of mature motor neurons. White arrows denote ChAT + motor neurons lacking TdTomato and thus Cre activity. Scale bar = 50 µm. **B** Weight of 26-week-old male BAC fxAR121; VChAT-Cre mice compared to singly transgenic BAC fxAR121 and control littermates, *n* = 9–14 mice/group. One-way ANOVA with post-hoc Tukey test, ****P < 0.0001. **C**) Weight of 26-week-old male BAC fxAR121; Hb9-Cre mice compared to singly transgenic and control littermates, *n* = 8–21 mice/group. One-way ANOVA with post-hoc Tukey test, *P < 0.05, ***P < 0.001, ****P < 0.0001. **D**) Representative images of spinal cord sections from symptomatic 9–10 month-old BAC fxAR121 mice and unaffected BAC fxAR121; Hb9-Cre mice stained for mutant human AR (white) and ChAT (green). White arrow denotes motor neurons with strong polyQ-hAR expression, while yellow arrows denote motor neurons with moderate expression. Scale bar = 50 µm. **E**) Quantification of motor neuron area in lumbar spinal cord of symptomatic 9–10 month-old BAC fxAR121 mice and unaffected BAC fxAR121; Hb9-Cre mice; *n* = 68–100 motor neurons/animal, 3–4 mice/group. Nested *t*-test, *P* = n.s. Error bars = s.e.m

Although we have shown that expression of mutant polyQ-AR in skeletal muscle is required for severe neuromuscular disease phenotypes to develop in SBMA model mice [4], the contribution of motor neuron expression of polyQ-AR to neuromuscular disease is yet to be directly addressed. To assess the relative contribution of motor neurons for driving neuromuscular decline in SBMA, we developed two different motor neuron Cre recombinase transgenic lines bred with BAC fxAR121 mice for comparison with rescued BAC fxAR121; HSA-Cre mice. We first used a line in which Cre recombinase is expressed from the promoter of vesicular acetylcholine transporter (VChAT) [15]. However, as Cre enzyme activity is active in only ~ 60% of motor neurons in VChAT-Cre mice [16], incomplete polyQ-AR excision could thus mask any potential phenotype rescue. Hence, we also used a second line, Hb9-Cre, with more complete motor neuron penetrance. In Hb9-Cre mice, the gene encoding Cre recombinase replaces one copy of *Hb9*, a transcription factor expressed by all motor neurons during development [17]. Crossing Hb9-Cre and VChAT-Cre mice with floxed TdTomato reporter mice confirmed that in VChAT-Cre; TdTomato bigenic mice, occasional motor neurons are not TdTomato + and thus did not undergo Cre-mediated recombination (Fig. 4A). In the case of bigenic Hb9-Cre; TdTomato mice, we did not detect a single instance of lack of TdTomato expression in ChAT + motor neurons (Fig. 4A), indicating highly efficient Cre-mediated excision using this Hb9-Cre line. We tracked body weight in male progeny obtained from crosses of both VChAT-Cre and Hb9-Cre with BAC fxAR121 mice, and we found that both male BAC fxAR121; VChAT-Cre and male BAC fxAR121; Hb9-Cre bigenic mice weighed much less than male littermate control mice at 26 weeks of age (Fig. 4B-C), in agreement with the weight loss observed in singly transgenic male BAC fxAR121 mice (Additional File 1: Figure S1). To determine if excision of the human polyQ-AR transgene in male BAC fxAR121; Hb9-Cre bigenic mice is as efficient as was observed in Hb9-Cre; TdTomato mice, we immunostained spinal cord sections from male BAC fxAR121; Hb9-Cre bigenic mice and age-matched male singly transgenic BAC fxAR121 mice, and confirmed that, as expected, motor neurons from male BAC fxAR121; Hb9-Cre bigenic mice do not display detectable AR expression, while motor neurons from male singly transgenic BAC fxAR121 mice exhibit AR expression in the nucleus, albeit variably (Fig. 4D). We also measured motor neuron area in spinal cord sections from end-stage male BAC fxAR121; Hb9-Cre bigenic mice and age-matched male singly transgenic BAC fxAR121 mice, and documented no improvement in the BAC fxAR121; Hb9-Cre mice (Fig. 4E).

### Male BAC fxAR121; Hb9-Cre mice display shortened survival and respiratory neuromuscular disease

**Fig. 5:**
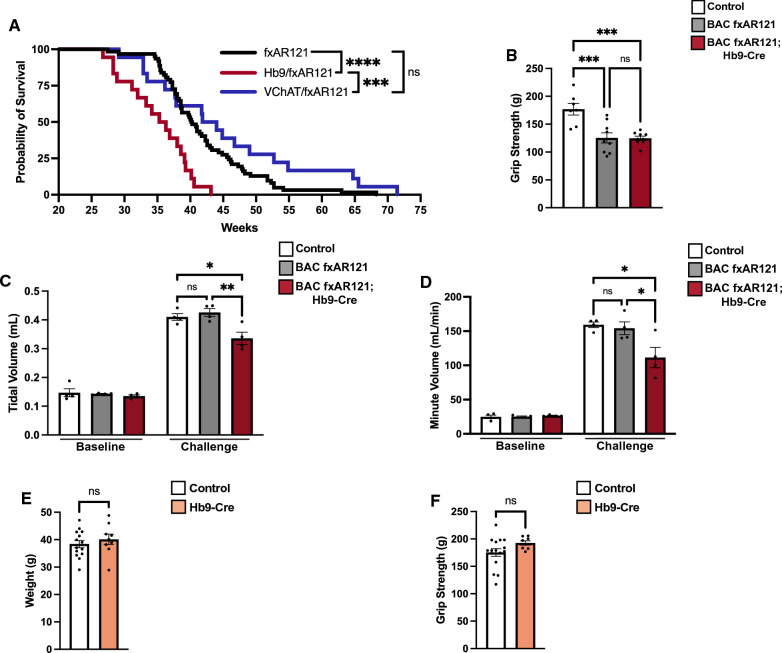
BAC fxAR121; Hb9-Cre motor neuron-rescued mice do not differ in weight or grip strength compared to BAC fxAR121 mice, but display worse respiratory function. **A** Kaplan–Meier survival analysis of male BAC fxAR121; VChAT-Cre, BAC fxAR121; Hb9-Cre, and singly transgenic BAC fxAR121 mice, *n* = 18–62 mice/group. Median survival is as follows: BAC fxAR121 mice = 40.3 weeks, BAC fxAR121; VChAT-Cre mice = 42.9 weeks, and BAC fxAR121; Hb9-Cre mice = 35.8 weeks. Log-rank Mantel-Cox test, ***P < 0.001, ****P < 0.0001. **B** Assessment of grip strength for 26-week-old BAC fxAR121 mice, BAC fxAR121; Hb9-Cre mice, and wildtype control mice, *n* = 8–21 mice/group. One-way ANOVA with post-hoc Tukey test, *P < 0.05, **P < 0.01. **C**, **D** Respiratory performance assessed by whole body plethysmography of pre-symptomatic 16-week-old male BAC fxAR121 mice, BAC fxAR121; Hb9-Cre mice, and wildtype control mice showing tidal volume (**C**) and minute volume (**D**), n = 4 mice/group. No differences were detected at baseline (room air), but significant differences were detected during challenge (hypoxia). One-way ANOVA with post-hoc Tukey test, *P < 0.05, **P < 0.01. **E** Weight of aged 16–20 month-old male wildtype and Hb9-Cre singly transgenic mice, *n* = 8–16 mice/group. Two-tailed *t*-test, *P* = n.s. **F** Grip strength of aged 16–20 month-old male wildtype and Hb9-Cre mice, *n* = 8–16 mice/group. Two-tailed *t*-test, *P* = n.s. Error bars = s.e.m

To further characterize disease phenotypes in male BAC fxAR121; VChAT-Cre and BAC fxAR121; Hb9-Cre bigenic mice, we compared their lifespans with singly transgenic male BAC fxAR121 mice, and we found no evidence for prolonged survival in either BAC fxAR121; VChAT-Cre nor BAC fxAR121; Hb9-Cre bigenic mice (Fig. 5A). Hence, elimination of human polyQ-AR expression from motor neurons did not improve lifespan in SBMA model mice. However, we unexpectedly noted that male BAC fxAR121; Hb9-Cre bigenic mice actually display significantly shortened survival times, as their median lifespan is ~ 4 weeks less than male singly transgenic BAC fxAR121 littermates (Fig. 5A). This occurs even though male BAC fxAR121; Hb9-Cre bigenic mice do not suffer worse neuromuscular disease in the periphery, as their grip strength is virtually identical to that of male singly transgenic BAC fxAR121 littermates (Fig. 5B). To better understand why male BAC fxAR121; Hb9-Cre bigenic mice display a further shortened lifespan, we performed more detailed phenotype analysis, including whole body plethysmography to assess critical respiratory function under ventilation challenge conditions that can expose subtle, early neuromuscular deficits. At the early, pre-symptomatic time point of 16 weeks of age, we evaluated male BAC fxAR121; Hb9-Cre bigenic mice, age-matched singly transgenic BAC fxAR121, and non-transgenic littermate controls by performing whole body plethysmography under baseline conditions (room air) and during a respiratory challenge (7% CO_2_ and 10% O_2_). We documented significant decreases in both tidal volume and minute volume in BAC fxAR121; Hb9-Cre mice when subjected to the respiratory challenge (Fig. 5C-D). These results indicate that neuromuscular dysfunction of the diaphragm, intercostal, and airway muscles is already underway in male BAC fxAR121; Hb9-Cre bigenic mice at this early age. As Hb9-Cre transgenic mice are haploinsufficient for Hb9 expression due to targeted transgenesis of the Cre-recombinase into one Hb9 allele, and homozygous Hb9-Cre are non-viable [18], it appears that Hb9 is critically required for motor neuron development and function. Hence, shortened survival of male BAC fxAR121; Hb9-Cre mice could reflect impaired motor neuron function due to reduced Hb9 expression, or could stem from complete elimination of human AR transgene expression, as polyQ-AR neurotoxicity is believed to involve both a gain of toxic function and a loss of normal AR function [19]. To examine the effect of loss of Hb9 function, we compared body weight and grip strength in aged Hb9-Cre transgenic mice and non-transgenic littermate controls, and we found that these read-outs were virtually identical (Fig. 5E-F).

## Discussion

The utility of mouse models of neurodegenerative disease for studying disease natural history, identifying pathogenic mechanisms, and evaluating therapies remains a contentious topic in the field. We, and others, have developed highly representative mouse models of SBMA, and have used these models to delineate a principal role for skeletal muscle expression of mutant polyQ-AR protein as a driver of SBMA motor neuron degeneration [4, 5, 20]. In human SBMA patients, electrophysiological studies have clearly documented reduced compound motor action potentials and estimated decreased motor unit numbers [21, 22], yet unlike patients with other lower motor neuron diseases, levels of neurofilament light chain—an established biomarker of ongoing degeneration—are not elevated in SBMA patients [23]. Here, through the parallel approach of excision of mutant *AR* from motor neurons using two different motor neuron-specific Cre drivers, we further validate a critical and primary role of polyQ-AR expression in skeletal muscle for driving SBMA neuromuscular decline, as male BAC fxAR121; VChAT-Cre and BAC fxAR121; Hb9-Cre mice do not display any improvement in lifespan or neuromuscular function. Despite these definitive findings, lingering questions remain as to the validity of SBMA model mice for faithfully recapitulating disease manifestations described in human patients. Although the BAC fxAR121 transgene harbors native regulatory elements to drive expression at variable, cell type-appropriate levels, one confounding issue is the apparently low-level expression of *AR* in motor neurons, as only ~ 5% of ChAT + motor neurons in the lumbar cord of our SBMA mice strongly co-stain for AR protein. As single-nucleus sequencing studies of mice revealed that *Ar* expression in motor neurons is relatively low [24, 25], it appears that our findings are not due to a technical artifact. Given this low expression level, it makes sense that excision of polyQ-AR from motor neurons did not confer any disease rescue in male BAC fxAR121; VChAT-Cre or BAC fxAR121; Hb9-Cre mice. It remains to be determined, however, if *AR* expression in human spinal cord motor neurons is as low as observed in model mice. Recent single-nucleus transcriptome profiling of human lumbar spinal cords from four adult males and three adult females does support the notion that *AR* expression in motor neurons is not high, as counts per million (CPM) for *AR* averaged from ~ 12 to 60 in the motor neuron cluster, with no clear separation between males and females, while *AR* CPMs ranged from ~ 50 to > 250 for a dorsal excitatory interneuron cluster [26]. Nonetheless, *AR* is still expressed at a detectable level in human motor neurons; hence, a role for polyQ-AR expression in human motor neurons as a contributor to the disease process in SBMA patients should not be dismissed.

An ongoing discussion in the field is whether emerging SBMA therapies should be delivered to the CNS or peripherally to skeletal muscle. Each approach has unique challenges, as CNS delivery will likely require invasive intrathecal injections, while peripheral administration will require very high dosages to effectively target skeletal muscle, which is an enormous organ accounting for ~ 40% of total body weight. Our findings in SBMA model mice add to existing clinical studies performed in human SBMA patients that strongly support the view that therapy delivery to skeletal muscle should be prioritized. Indeed, in human SBMA patients, plasma levels of neurofilament light chain, a well-established biomarker of neurodegeneration used in the devastating motor neuron disease amyotrophic lateral sclerosis, are not elevated, while serum levels of creatine kinase, a marker of cell autonomous muscle damage, are robustly elevated [27]. Not only does this latter finding provide evidence of primary muscle dysfunction in SBMA, but also indicates that suitable biomarkers for tracking muscle involvement in SBMA should be feasible, which will facilitate drug development and clinical trial testing. Further evidence supporting a pathogenic role for skeletal muscle in SBMA comes from a case report delineating how an exercise intervention consisting of 15 weeks of functional resistance training targeting skeletal muscle led to marked improvement in strength and balance in a SBMA patient [28]. Finally, a SBMA Phase 2 clinical trial of peripherally administered BVS857, a synthetic insulin-like growth factor 1 mimetic, prevented a decline in thigh muscle volume in treated patients vs. placebo [29]. Unfortunately, immunogenicity of BVS857 has precluded further investigation. These reports, taken together with our current finding that genetic excision of mutant AR from motor neurons does not rescue neuromuscular decline in SBMA mice, indicate that therapy development for SBMA patients should focus on peripheral interventions targeting skeletal muscle. However, a combinatorial approach with delivery to the CNS and to skeletal muscle remains a highly appealing option since it is impossible to exclude a cell-autonomous contribution of polyQ-AR to motor neuron demise in human patients.

Another important aspect of SBMA disease natural history gaining in recognition is the presence of non-CNS pathology. Non-alcohol-associated fatty liver disease is a nearly universal clinical feature of SBMA, and it is usually accompanied by additional signs of metabolic dysfunction, such as hyperlipidemia and insulin resistance [6, 7, 22]. Cardiac involvement in SBMA is less well-characterized, but a number of reports have documented Brugada-type abnormal echocardiogram patterns and hypertrophic-like cardiomyopathy in a subset of SBMA patients [8, 9], although some have argued that these minor cardiac abnormalities are due to unrelated causes, such as aging [30]. Since unaddressed cardiac dysfunction can be life-threatening, further studies are warranted and led us to evaluate SBMA model mice for liver and heart disease. To facilitate detection of hepatic and cardiac pathology, we focused on “muscle-rescued” BAC fxAR121; HSA-Cre transgenic mice, reasoning that their extended lifespans would provide more time for systemic phenotypes to emerge. Analysis of aged male BAC fxAR121; HSA-Cre bigenic mice revealed prominent evidence of non-alcohol-associated fatty liver disease, cardiomegaly, and ventricular wall thinning. In fact, mutant AR-driven cardiomyopathy is the most likely explanation for the sudden and unexpected demise of aged BAC fxAR121; HSA-Cre mice. Our discovery of significant hepatic disease and cardiac abnormalities in SBMA model mice underscores the need to evaluate human SBMA patients for signs of liver disease and heart disease, as treatment of these conditions could improve the health, quality of life, and survival of this patient population.

While SBMA model mice are revealing aspects of disease natural history with implications for clinical care of human patients, one feature that is unique to BAC fxAR121 and other representative mouse models is markedly shortened lifespan, as most SBMA patients live a normal lifespan, with only some patients succumbing to their disease when middle-aged or elderly. Why do SBMA mice often display premature death? One likely factor is the use of supraphysiological repeat expansions of > 100 CAGs to model disease, as the largest AR CAG repeat expansion identified to date in a SBMA patient was 68 CAGs [31]. Another issue is the specialized biology of male mice who possess the ability to urinate on demand for behavioral reasons and thus have extensive bulbocavernosus (BC) and levator ani (LA) musculature, which is known to abundantly express AR [32]. For this reason, we, and others, have noted that male SBMA mice with BC/LA muscle dysfunction suffer from post-renal obstruction leading to accumulation of urine in the bladder and consequently kidney disease [33]. In this study, we noted that male BAC fxAR121; Hb9-Cre bigenic mice actually display even shorter survival than singly transgenic male BAC fxAR121 mice, and by performing whole body plethysmography, we found that BAC fxAR121; Hb9-Cre mice develop respiratory muscle dysfunction at an earlier timepoint. As Hb9-Cre recombinase is highly efficient at excision of loxP flanked targets in motor neurons, it appears that very little to no human AR protein is expressed in motor neurons of male BAC fxAR121; Hb9-Cre bigenic mice. Hence, it is possible that loss of AR normal function due to complete elimination of AR transgene protein in BAC fxAR121; Hb9-Cre mice could be contributing to the worsened respiratory muscle function, as Hb9-Cre mice do not show evidence of impaired neuromuscular function, ruling out loss of Hb9 function as a likely explanation, though further research to clarify the basis of this defect is warranted. In conclusion, our studies illustrate that mouse models of CAG-polyQ repeat disorders remain a valuable tool for deconstructing disease pathobiology and can provide unexpected insights into disease natural history as well as the cellular and molecular basis of the neurodegeneration.

## Materials and methods

### Animal studies

Animal studies were conducted under protocols approved by the Institutional Animal Care and Use Committee of the University of California San Diego, Duke University, and University of California Irvine. The strains used in this study were all previously described, including the SBMA model strain BAC fxAR121 [4], skeletal muscle Cre driver HSA-Cre [34] (JAX strain #006149), motor neuron Cre drivers VChAT-Cre [15] (kind gift of Dr. Don Cleveland, UCSD) and Hb9-Cre [17] (JAX strain #006600), and TdTomato flox Cre reporter [35] (JAX strain #007909). Due to the sex-specific manifestation of SBMA, only male mice were used. For survival analysis, mice were counted that were either found dead or had reached a humane endpoint in which they were below 22 g in weight, had enlarged bladders that could be readily detected by palpitation which did not result in assisting with urination (i.e., end-stage urinary blockage), and exhibited limited movement and response to stimuli. For grip strength analysis, mice were grasped by the tail, lowered toward a grid attached to a force meter (Bioseb #BIO-GS3) until they held on with all 4 paws, and gently pulled until they could no longer hang on. This was repeated 6 times per mouse, then the lowest and higher values were excluded, and the remaining 4 values were averaged for the final reading. Tests in which the mouse bit the grid or did not hold on with all 4 paws were not counted. Whole body plethysmography was performed as previously described [36]. Briefly, mice were placed into plethysmography chambers designed for small animals (Buxco) and habituated at normoxia (FiO2 0.21/N2 balance) for at least 1.5 h. After a 5-min baseline reading, mice were exposed for 10 min to a respiratory challenge environment (FiCO2 0.07/FiO2 0.10/N2 balance). Respiration data was acquired and analyzed by FinePoint software.

### BAC fxAR121 transgene analysis

Copy number of transgenic human androgen receptor gene were determined by Taqman Copy Number Assay (Thermo Fisher Scientific) using probes against human *AR* (Hs00052407) normalized to mouse *Tert* (#4,458,368) according to manufacturer instructions. Primers to detect polyQ length in genomic DNA isolated from fxAR121 animals born in 2010, 2014, and 2020 were: Forward ACCTCCCGGCGCCAGTT and Reverse TGCTGCTGCCTGGGGCTA. Predicted molecular weight (assuming 121 CAG repeats) was 417 bp.

### Western blot analysis

Protein samples from skeletal muscle were prepared by first snap freezing dissected tissues in liquid nitrogen, then homogenizing tissues in RIPA buffer supplemented with protease and phosphatase inhibitors (Thermo Fisher Scientific) using a Qiagen Tissuelyser LT and Qiashredder column. Soluble protein was quantified by Qubit Protein Assay and prepared according to manufacturer instructions (Thermo Fisher Scientific). Proteins were separated on 4–20% Stain-Free TGX gels (Bio-Rad), transferred to PVDF, and blocked in 5% non-fat dry milk in TBST (1X TBS with 0.05% Tween-20) for 1 h at RT. Membranes were incubated with primary antibody recognizing human and mouse AR (Abcam #ab108341) for 48 h, washed, then incubated with anti-rabbit secondary antibody conjugated to horseradish peroxidase for 1 h. Signal was detected with SuperSignal West Dura (Thermo Fisher Scientific) and imaged on a Bio-Rad ChemiDoc system. Densitometry analysis was performed using Image Lab software and normalized to total protein as detected by stain-free technology (Bio-Rad).

### Histology

For histological analysis, tissues were dissected and fixed overnight in 4% (spinal cord and liver) or 2% (skeletal muscle and heart) paraformaldehyde in 1X PBS, sunk in 30% or 20% sucrose, respectively, embedded in O.C.T. (Sakura), frozen in liquid nitrogen-cooled 2-methylbutane, and stored in −80 °C. 20 (spinal cord) or 10 (muscles, liver, heart) micron-thick sections were cut on a Leica CM3050S cryostat and mounted directly on Superfrost Plus Slides (Fisherbrand). For immunostaining, sections were blocked with 4% BSA/0.01% Triton X-100 in 1X PBS for 1 h, then incubated with primary antibodies for 2 days at 4 °C (for ChAT) or overnight at room temperature (for all others). Primary antibodies were against laminin (Sigma #L0663, 1:250 dilution), collagen I (Abcam #ab21286, 1:250 dilution), sarcomeric alpha-actinin (Sigma #A7811, 1:500 dilution), human-specific AR (Cell Signaling Technology #5153, 1:100 dilution), and ChAT (Millipore #A144P, 1:100 dilution). Secondaries used were raised in donkey against rat, rabbit, mouse, or goat and conjugated to Alexa Fluor 488, 555, or 647 Plus (Thermo Fisher Scientific). Histological stains were performed using Oil Red O Stain Kit (Abcam #ab150678) and Picrosirius Red Stain Kit (Abcam #ab150681) according to manufacturer instructions. Images were acquired using a Nikon A1R confocal microscope or ECHO Revolution microscope and analyzed using Nikon Elements software. For measurements of cardiac tissue, one section was chosen per animal corresponding to the thickest part of the mid-level, containing papillary muscles and lacking atria. For each metric, 5 measurements were taken manually using FIJI and averaged.

### Statistical analysis

All statistical analyses was performed using Prism 9.0 (GraphPad). Data are shown as mean ± SEM. Differences between groups were considered significant at *P* < 0.05 and are indicated on the figure graphs by conventional asterisk formatting (* *P* < 0.05, ** *P* < 0.01, *** *P* < 0.001, and **** *P* < 0.0001).

## Supplementary Information

**Additional file 1**. **Figure S1**: Characterization of body weight of male wildtype and HSA-Cre only controls compared to singly transgenic BAC fxAR121 and muscle-rescued BAC fxAR121;HSA-Cre mice. A) Weight of individual animals of each genotype plotted as a function of time and fitted with a simple linear regression. Slopes and R squared values are as follows: wildtype, HSA-Cre, HSA/fxAR121, fxAR121. B) Gross anatomical analysis upon dissection of agedmale mice showing robust inguinal, epidydimal, and mesenteric white adipose deposits in control miceand the complete absence of these deposits in BAC fxAR121; HSA-Cre mice.
