# Supplementary figures and images for: X-linked SBMA model mice display relevant non-neurological phenotypes and their expression of mutant androgen receptor protein in motor neurons is not required for neuromuscular disease
Source: Acta Neuropathol Commun. 2023 Jun 2;11:90. doi: 10.1186/s40478-023-01582-1 (PMC10239133; doi:10.1186/s40478-023-01582-1)

**A**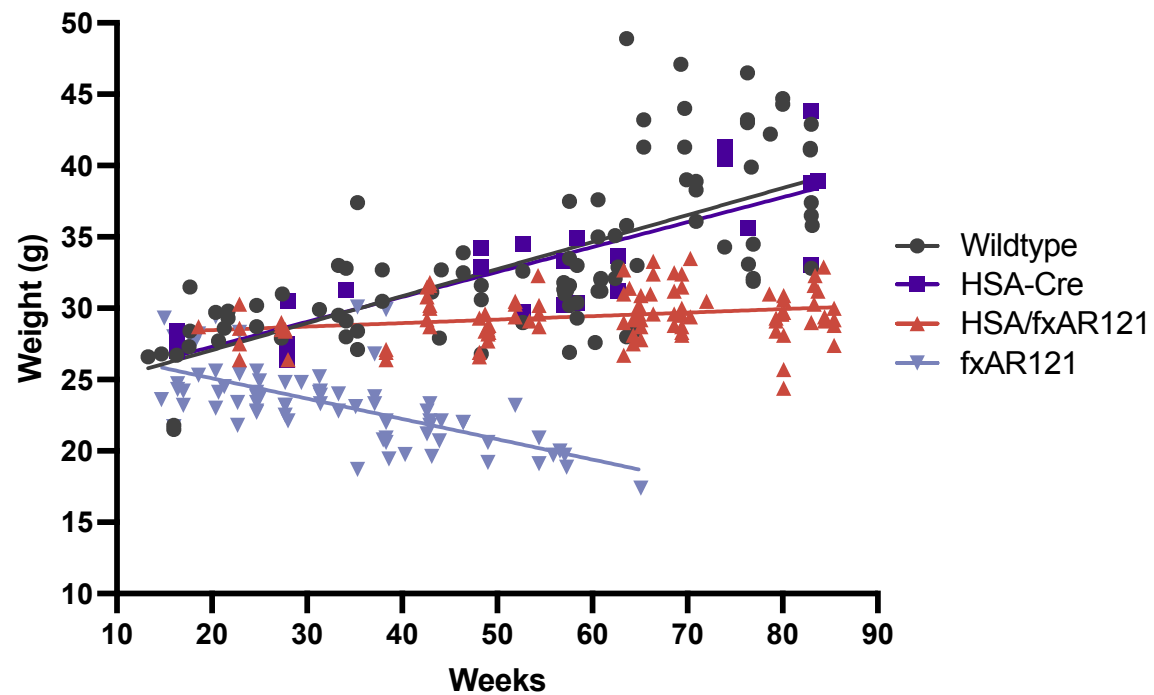**B**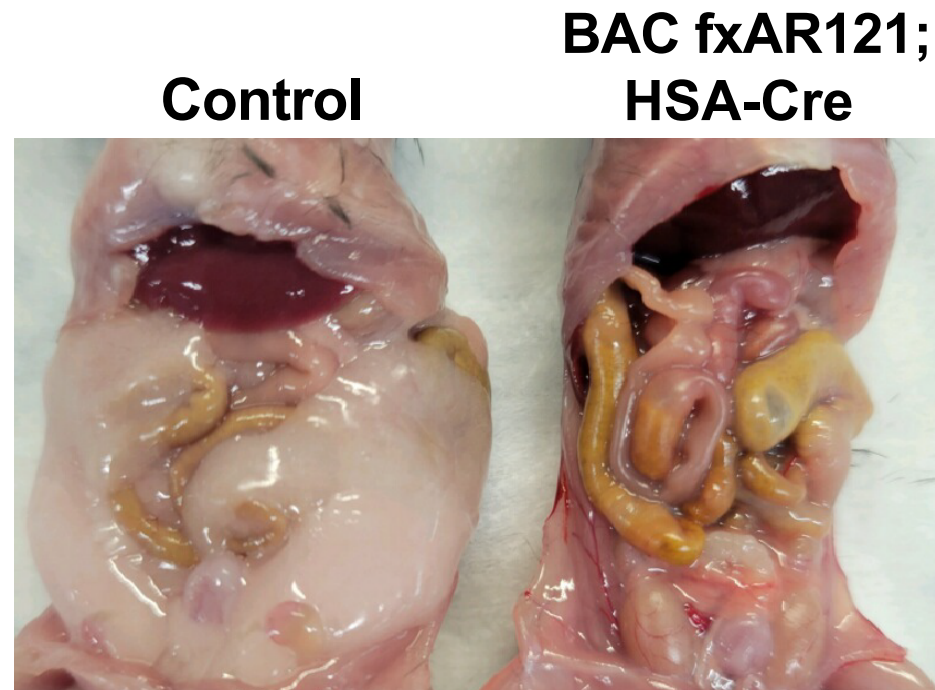

Supplement: Supplementary file 1 — Additional file 1. Figure S1: Characterization of body weight of male wildtype and HSA-Cre only controls compared to singly transgenic BAC fxAR121 and muscle-rescued BAC fxAR121;HSA-Cre mice. A) Weight of individual animals of each genotype plotted as a function of time and fitted with a simple linear regression. Slopes and R squared values are as follows: wildtype, HSA-Cre, HSA/fxAR121, fxAR121. B) Gross anatomical analysis upon dissection of agedmale mice showing robust inguinal, epidydimal, and mesenteric white adipose deposits in control miceand the complete absence of these deposits in BAC fxAR121; HSA-Cre mice. [file 40478_2023_1582_MOESM1_ESM.pdf]
